# The Challenges of Vaccine Development against Betacoronaviruses: Antibody Dependent Enhancement and Sendai Virus as a Possible Vaccine Vector

**DOI:** 10.1134/S0026893320060151

**Published:** 2020-09-04

**Authors:** T. A. Zaichuk, Y. D. Nechipurenko, A. A. Adzhubey, S. B. Onikienko, V. A. Chereshnev, S. S. Zainutdinov, G. V. Kochneva, S. V. Netesov, O. V. Matveeva

**Affiliations:** 1 Sendai Viralytics, 117261 Acton, MA USA; 2 Engelhardt Institute of Molecular Biology, Russian Academy of Sciences, 119991 Moscow, Russia; 3 George Washington University, 20052 Washington, DC USA; 4 Department of Military Field Therapy, Kirov Military Medical Academy, 194044 St. Petersburg, Russia; 5 Institute of Immunology and Physiology, 620049 Yekaterinburg, Russia; 6 State Research Center of Virology and Biotechnology “Vector”, 630559 Koltsovo, Russia; 7 Department of Natural Sciences, Novosibirsk State University, 630090 Novosibirsk, Russia; 8 Biopolymer Design, 117281 Acton, MA USA

**Keywords:** SARS-CoV-2, SARS-CoV-1, COVID-19, antibody-dependent enhancement, ADE, vaccine vector, Sendai virus, murine respirovirus, conservative antigenic determinants

## Abstract

To design an effective and safe vaccine against betacoronaviruses, it is necessary to use their evolutionarily conservative antigenic determinants that will elicit the combination of strong humoral and cell-mediated immune responses. Targeting such determinants minimizes the risk of antibody-dependent enhancement of viral infection. This phenomenon was observed in animal trials of experimental vaccines against SARS-CoV-1 and MERS-CoV that were developed based on inactivated coronavirus or vector constructs expressing the spike protein (S) of the virion. The substitution and glycosylation of certain amino acids in the antigenic determinants of the S-protein, as well as its conformational changes, can lead to the same effect in a new experimental vaccine against SARS-CoV-2. Using more conservative structural and accessory viral proteins for the vaccine antigenic determinants will help to avoid this problem. This review outlines approaches for developing vaccines against the new SARS-CoV-2 coronavirus that are based on non-pathogenic viral vectors. For efficient prevention of infections caused by respiratory pathogens the ability of the vaccine to stimulate mucosal immunity in the respiratory tract is important. Such a vaccine can be developed using non-pathogenic Sendai virus vector, since it can be administered intranasally and induce a mucosal immune response that strengthens the antiviral barrier in the respiratory tract and provides reliable protection against infection.

## INTRODUCTION

At the end of 2019, the new severe acute respiratory syndrome betacoronavirus SARS-CoV-2 (severe acute respiratory syndrome coronavirus 2) caused an infectious disease in China called COVID-19, which then spread and grew into a global pandemic. The creation of an efficient vaccine directed at the conservative antigens of betacoronavirus will help limit the spread and prevent COVID-19 or at least attenuate its progression.

The vaccine approaches for COVID-19 are extremely diverse. This review analyzes the problems encountered in creating vaccines targeting SARS-CoV-2. In addition, non-pathogenic viral vectors for the expression of antigenic determinants of this virus have been examined. We present arguments in favor of the application of the Sendai virus-based vector for vaccine creation.

## THE CHALLENGES OF DEVELOPING AN EFFECTIVE AND SAFE COVID19 VACCINE

A serious problem with coronavirus vaccines can be a secondary immune response leading to antibody-dependent enhancement of infection (ADE) and the development of respiratory distress syndrome. It is important to find out as early as possible that the experimental vaccine is not priming ADE development, although this is not an easy task. For example, the effect of ADE was detected during mass immunization of children in the Philippines with a vaccine against Dengue virus (Dengvaxia) manufactured by Sanofi Pasteur (France) [1].

### The Phenomenon of Antibody-Dependent Infection Enhancement

**Fig. 1.**
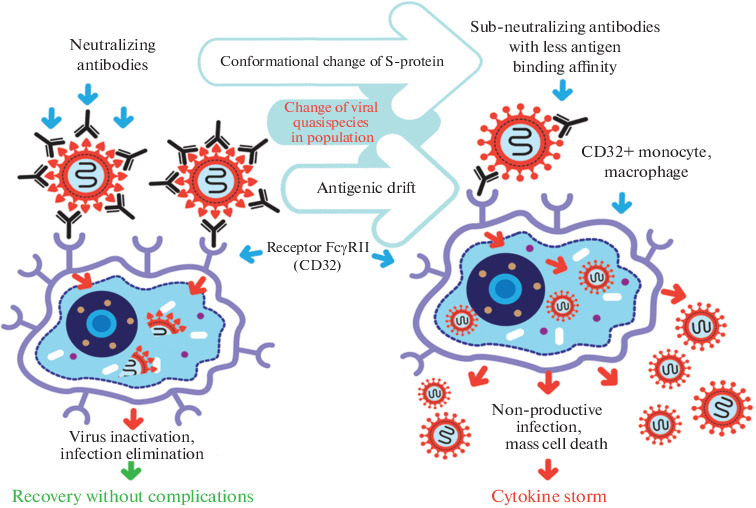
Scheme of antibody-dependent infection enhancement (ADE) for SARS-CoV-1. On the left, a scenario of the correct immune response is shown, when specific neutralizing and protective antibodies contribute to the elimination of the virus from the body. According to this scenario viruses are phagocytosed as stable antigen–antibody complexes and destroyed by macrophages or other immune cells. On the right is an immunopathology scenario that occurs when the antigen of the virus changes and, because of this change, IgG antibodies form imperfect complexes with the virus. The unstable antibody–virus complex binds to the FсγRII receptor of immune cells and is absorbed by these cells. Further, inside the cell, the virus leaves the endosome, already without the antibody, and begins the replicative cycle [5, 10, 12].

The ADE phenomenon has been described for various viruses [2, 3], including coronaviruses [4–8]. Figure 1 illustrates the efficient “correct” handling of the virus–antibody complexes by immune cells compared to pathological infection aggravated by ADE. In the case of ADE, virus-specific IgG antibodies form non-stable complexes with the virus and after binding to FcγRII receptors expressed by some immune cells [9] facilitate infection of these cells [2, 5, 10, 11].

The virus internalized by monocyte or macrophage in a stable complex with antibodies cannot escape and is usually destroyed. The virus elimination promotes host recovery—as shown on the left side of Fig. 1. However, in the case of ADE, the virus frees itself from the less stable complex with antibody and starts the replicative cycle inside the immune cell, as shown in the right side of Fig. 1.

It has been demonstrated for SARS-CoV-1, that virus-specific S-protein antibodies can facilitate entry of the virus into host B-cells [13] and macrophages [7]. Antibodies promote virus attachment and entry into the immune cell, where it starts to replicate without production of viable virions [7]. This nonproductive infection can be due to inability of macrophages to express serine proteases required for the virion activation. It cannot be excluded though that produced inactive virions can get activated and become infectious during penetration into other host cells, which express membrane associated proteases (like TMPRSS2) necessary for the virion activation. However, even non-productive infection can lead to massive cell death of macrophages and other immune cells carrying FcγRII receptors, which can aggravate the course of the disease.

Similar to SARS-CoV-1 and MERS-CoV, SARS-CoV-2 might infect FcγRII-bearing immune cells (such as monocytes, macrophages, B-cells as well as some types of dendritic cells) and promote ADE.

### Antigenic Diversity of S-Protein Can Promote ADE of Betacoronaviruses Infection

**Fig. 2.**
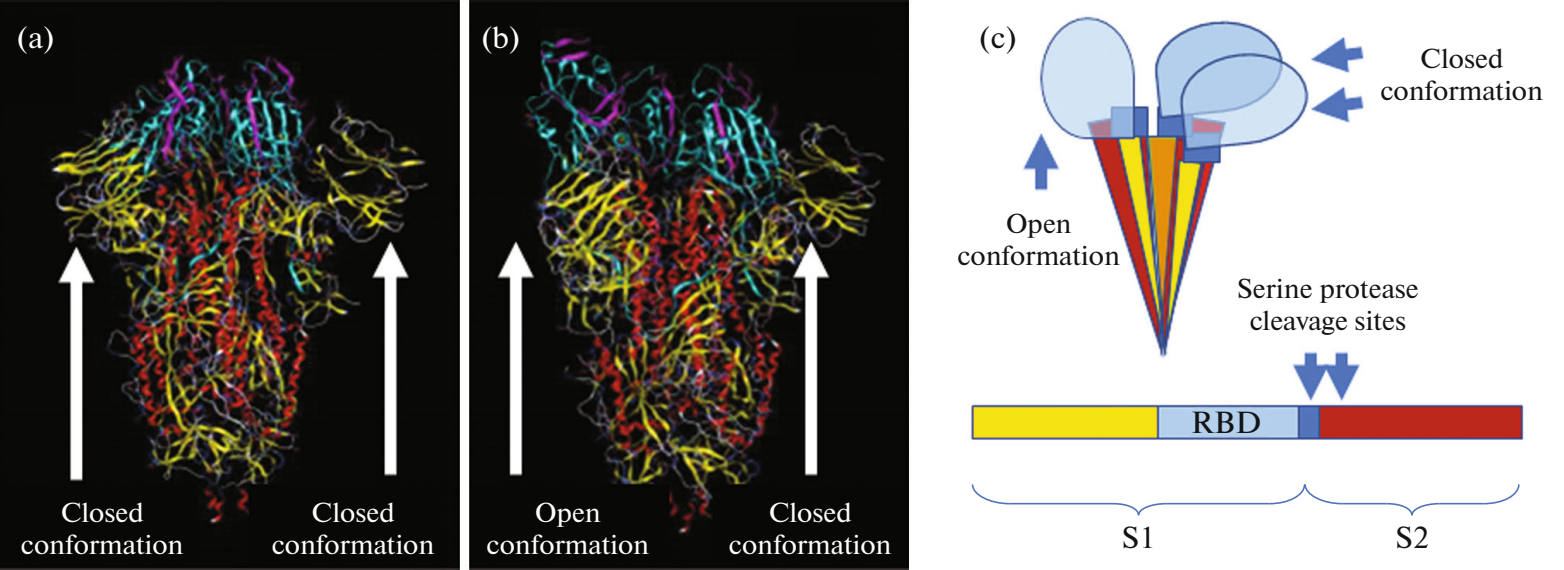
S-protein conformations in the homotrimer. (a) All S1 subunits are in a closed conformation. (b) One subunit is in an open conformation, and one is in a closed conformation. (c) Conformations of the S-protein in the trimer and the protein domain structure are shown schematically. The receptor-binding domain (RBD, blue), together with the N-terminal domain (yellow), is part of the S1 subunit. In the S1 subunit (blue) there is a proteolytic cleavage site for furin, and within the S2 subunit (brown) is a TMPRSS2 protease cleavage site [15]. Images are taken from the PDB database [16].

The Spike protein (S-protein) on the surface of the SARS-CoV-2 virion forms a trimer, each of the three molecules of which consists of two subunits: S1 and S2 [14]. Figure 2 schematically shows this protein conformations and subunits.

The N-terminal domain of S1 subunit is responsible for the virus–receptor binding and a C-terminal S2 subunit is responsible for the viral envelope–cellular membrane fusion. S1 subunit includes Receptor Binding Domain (RBD) and can exist in two different conformations—open and closed [17, 18]. Cryo-electron microscopy revealed that most often one of the S1 subunits is in an open conformation, and the other two are in a closed conformation [14]. As seen from Fig. 2, when the S-protein conformation changes, some of its antigenic determinants will also inevitably undergo rearrangements. Thus, the ADE phenomenon in SARS-CoV-2 theoretically may be a result of the antigenic variability of the S-protein. This variability might be caused by the amino acids’ substitutions in its S1 subunit [19], by different glycosylation of amino acids residues [20], and, in addition, by the protein conformational mobility.

**Fig. 3.**
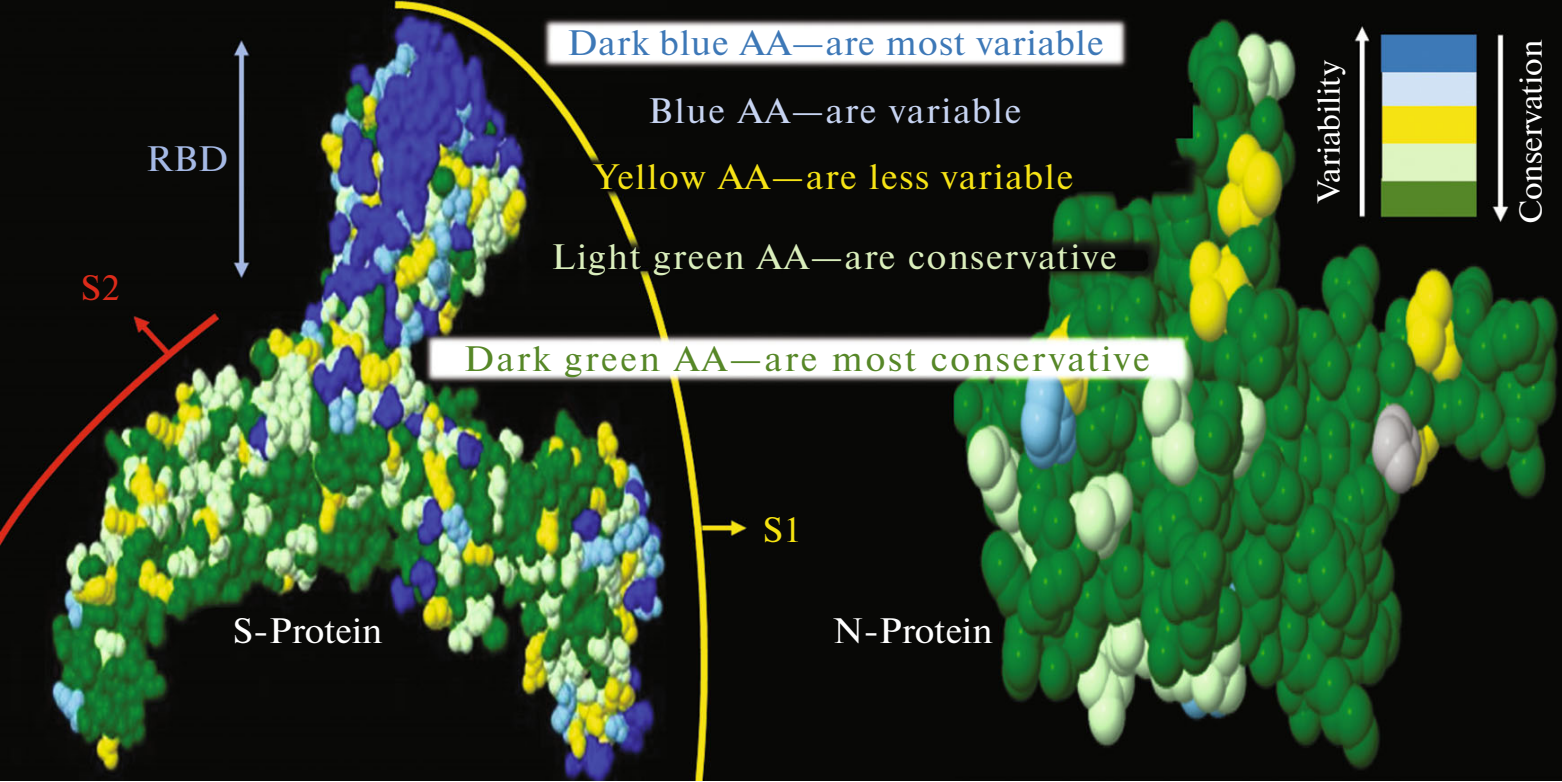
Models of S- and N-protein structures of betacoronaviruses. Conservative and variable amino acids (AA) are shown in different colors. Protein structures reproduced from the preprint [21]. It is assumed that the high variability of the S-protein is due to its surface exposure on the virion and, as a result, fast antigenic drift under the pressure of immune surveillance. In contrast the N-protein, which is mainly located inside the virion and less visible for humoral immunity is more conservative.

In several studies that were done with the SARS-CoV-1, MERS-CoV viruses ADE was shown to be a result of the changing immunodominant determinants of the S-protein [6, 8]. Based on the analysis of these and other studies along with genomic sequences of viruses, Ricke et al. [21] hypothesized that SARS-CoV-1, MERS-CoV and SARS-CoV-2 viruses use a universal mechanism leading to ADE infection of CD32+ immune cells. The analysis of the mutational variability of betacronaviruses proteins seems to be very important in this context (see Fig. 3).

By comparing different isolates of SARS-CoV-2 and some other betacoronaviruses Ricke et al. [21] demonstrated very high variability of the S-protein in contrast to the N-protein and other viral proteins. Similar high variability of antibody exposed domains of S-protein in betacoronaviruses was also demonstrated in another study [18]. These studies show positions of amino acids that are vulnerable to substitutions in a process of antigenic drift. These substitutions can lead to some changes of S-protein antigenic determinants and consequent ADE.

Some SARS-CoV-2 isolates have aspartic acid at position 614 in S-protein and some of them have glycine [22, 23]. Position 614 is close to RBD, and substitution of negatively charged aspartic acid for neutral glycine may play a key role in ADE due to altered antigenic determinants of S1 subunit [22, 24]. Two hypotheses have been proposed to explain this phenomenon [22]. The first hypothesis is that amino acid substitution can cause ADE directly, due to a change in the binding constant of an antibody to the antigenic peptide, which includes amino acid residue 614. The authors noticed that the antigenic peptide LYQDVNC is identical between SARS-CoV-1 (S597–603) and SARS-CoV-2 (S611–617). They rationalized that antibodies to this peptide, theoretically, can cause ADE for SARS-CoV-2 because they cause ADE for SARS-CoV-1.

The second hypothesis is that the replacement of the amino acid at position 614 can lead to ADE, promoting the transition of the open S-protein conformation to closed. After replacement of aspartic acid with glycine at position 614—the hydrogen bond between the S1 and S2 subunits disappears. Weakening of hydrogen bonding between two subunits can trigger S‑protein conformational change, that can cause decrease in binding constant of antibody–virus complex that can result in ADE [22].

In addition, ADE theoretically can be caused by SARS-CoV-2 antigen changes due to glycosylation pattern variation of the S-protein molecule. Watanabe et al. identified 22 potential glycosylation sites in this protein [20]. It is known that carbohydrate chains of proteins impede the binding of antibodies to epitopes and thereby promote the formation of low-affinity antibody–virus complexes that might promote ADE. On the other hand, the carbohydrate chains themselves can be part of antigenic determinants, and their absence in the protein will lead to a decrease in the binding affinity of the antibody to the antigen promoting ADE risk.

When a new host is infected, the infection occurs not with one variant of the virus, but with a whole population of genetically closely related variants that resulted from mutations, happened during the virus replication in previous hosts [25]. This population is called quasi-species. The concept of quasi-species helps to understand that for escape from the immune surveillance of a new host due to a change in antigenic determinants, SARS-CoV-2 does not have to acquire new mutations, it can take advantage of existing ones in its quasi-species population. One can hypothesize that some of these viral virus variants with predominantly open S-conformation get easily neutralized by antibodies of the new host. Perhaps some other variants of the virus with more closed conformation of the protein and weaker affinity for antibodies are not getting neutralized. Moreover, they gain an evolutionary advantage via infection of CD32+ immune cells promoting ADE.

### Consideration of S-Protein Antigenic Variability for a Vaccine Design

By comparing different isolates of SARS-CoV-2 virus with other betacoronaviruses high variability of antibody exposed domains of S-protein was demonstrated in at least two studies [18, 21]. In addition, the some amino-acid substitutions in this protein [19], and its variable glycosylation pattern were also demonstrated [20]. A vaccine created based on one variant of the S protein of SARS-CoV2 might induce the production of antibodies with high affinity to the vaccine antigen, but lower affinity for a circulating strain that has already undergone antigenic drift, which changed this protein. Therefore, that vaccine might lose its protective power. Moreover, the antibody–virus complex might take on the role of a “Trojan horse” making it easier for the virus to infect the host’s monocytes or macrophages and other CD32+ immune cells. The same scenario is possible with a primary infection during its development in the body due to changes in viral epitopes under the pressure of immune surveillance. It is also possible in case of a secondary infection with a mutated variant of the virus.

### ADE and Respiratory Distress Syndrome

Animal model studies on viral infections caused by SARS-CoV-1 and MERS-CoV demonstrated significant lung damage associated with ADE [4–8, 26]. Furthermore, there are studies indicating that class IgG antibodies against SARS-CoV-1 S-protein antigens induce a severe macrophage-mediated damage of lungs both in humans and in primates [27]. In rabbits used as an animal model of the disease caused by SARS-CoV, it has been shown that ADE can develop upon reinfection. Thus, in animals intranasally infected with SARS-CоV pulmonary pathology characterized by viremia and severe pneumonia. When re-infected with SARS-CoV, despite the presence of antibodies, the animal’s lung damage was even more severe than during the primary infection [27].

Infection with viruses SARS-CoV-1 [28] or MERS-CoV [29] caused more severe pneumonia in vaccinated animals, despite the high level of specific neutralizing antibodies. Interestingly, animal studies on the effectiveness of recombinant or inactivated virus vaccination lead to the same negative results [28, 30].

Some researchers have already demonstrated the absence of the ADE effect promoted by vaccination against SARS-CoV-2 virus in animal models and in cell cultures [31]. Using virus-like particles based on S-protein expressed in a retroviral construct, the authors showed that animal serum after immunization with a recombinant RBD fragment of S-protein prevents the penetration of virus-like particles into cells expressing antibody-binding macrophage and monocyte receptors—FcγRII (CD32). It should be noted that in this work, the natural process of viral infection was modeled only partially since it does not reproduce the whole variety of natural sequences and conformations of SARS-CoV-2 S-protein.

The ADE phenomenon, mediated by antibodies to the full-length S-protein SARS-CoV-1, was observed in primates. Although vaccination reduced the viral load following subsequent SARS-CoV-1 infection, the presence of IgG antibodies to the S protein in immunized macaques significantly increased inflammatory lung damage [27]. In humans, the immunodominant SARS-CoV-1 S-protein epitope induced the production of both neutralizing antibodies and antibodies enhancing the infection [27].

### ADE as a Possible Cause of Pathogenesis of SARS and COVID-19

Several research groups hypothesized that the pathogenesis of SARS and COVID-19 diseases is related to ADE [21, 22, 24]. The infection of CD32+ cells is assumed to be a key step in the development of the COVID-19 disease and its progression from mild to severe form. ADE can account for the observed impairment of immunity regulation, including apoptosis of immune cells leading to the development of the T-cell lymphopenia, an inflammatory cascade, as well as a cytokine storm.

It was reported [12] that expression of two types of receptors FcγRIIa and FcγRIIb (but not FcγRI or FcγRIIIa), induces ADE in SARS-CoV-1 infected cells of the human immune system. While observing SARS patients, Yuan et al. [32] found that the severity of the disease correlates with FcγRIIa’s allelic polymorphism; the disease was more severe in patients with FcγRIIa isoforms that bind to both IgG1 and IgG2 than in patients with FcγRIIa isoforms that bind exclusively to IgG2.

### ADE as a Cause of Severe Forms of COVID-19 that Prevail among Older Patients

Antibodies are produced slower in elderly people due to immunosenescence, and by the time the antibody titer reaches the level necessary to neutralize the virus, antigenic determinants of the pathogen have time to evolve. This can occur due to either direct mutations or activation of a new quasispecies different from the original dominant one. In this case, the neutralizing antibodies developed towards original antigenic determinants might start forming unstable complexes with the changed antigens and “drag in” the virus into monocytes and macrophages, where it can start to replicate. As a result, a generalized infection and a cytokine storm might develop.

Indeed, the titer of IgG antibodies against the S‑protein of SARS-CoV-2 virus correlates with age and severity of the disease in hospitalized patients: older patients are more likely to have higher antibody titers and more serious illness [33]. In addition, the levels of antibodies correlated significantly with the level of lactate dehydrogenase, the marker of inflammation [34] and acute myocardial injury [35].

The hypothesis suggesting that some variants of anti-S-protein antibodies may be harmful is also supported by the following observation. A comparative analysis of the specific humoral response in SARS-CoV-1 patients indicated that on the 15th day of the disease, the level of antibodies against the S-protein was significantly higher in patients who subsequently died than in those who subsequently recovered [36, 37].

### Antigenic Imprinting and Pathogenesis of COVID-19

The hypothesis put forward by J. Tetro [38] is associated with the phenomenon of antigenic imprinting [39] and is based on the possible immunological cross-reactivity between seasonal low pathogenic coronaviruses and SARS-CoV-2. Long-living memory cells remember all specific pathogens encountered during the primary infection and provide protection against subsequent infections by closely related pathogens. Memory B cells produce antigen-specific antibodies as a reaction to specific epitopes on the surface of viruses. When a new infection arises, the reaction of memory B cells to old antigens from the previous infection is faster than the reaction of naive B cells, which start producing antibodies against new antigens [39].

Antigenic imprinting accelerates the immune response; this is its main role for effective and rapid infection control. However, this phenomenon also has a negative side. During recall responses, memory B cells developed during seasonal low-pathogenic coronaviruses infections are more easily reactivated than are their naive counterparts and stimulate the production of corresponding antibodies. It is possible that such antibodies will have a reduced affinity for the epitopes of the new virus and therefore stimulate a weak immune response and/or induce ADE. As a result of ADE, massive death of immune cells can occur, and this process develops very quickly and in the early stages of infection—even before the body begins to produce enough virus-specific protective antibodies. Since the pool of memory B-cells capable of producing antibodies to previous infections, including seasonal and low-pathogenic coronaviruses, is likely to increase with age, antigenic imprinting may be the reason why COVID-19 is severe in older people. At the same time, due to the rapid response of the immune system to the pathogen, antigenic imprinting developed for seasonal coronaviruses can either prevent or alleviate the course of COVID-19. Most likely, the development of the disease in one form or another depends both on the individual characteristics of all systems of the human body and on the repertoire of pathogens with which the immune system has already encountered.

## IMMUNE RESPONSE IN COVID-19 PATIENTS

### Humoral Immune Response

The antibody responses most likely play an important role in the SARS-CoV-2 clearance and patients’ recovery. Thus, the virus-specific antibodies can be detected within three weeks in 100% of a few hundred of COVID-19 patients that were admitted to the hospital in China [40]. It was shown that IgG and IgM antibodies target primarily the S-protein [41]. Its RBD and N-terminal domains of the S1 subunit most frequently trigger the production of the virus neutralizing antibodies [42, 43]. In many patients N-protein is also a target for antibodies that are produced in high levels [44].

Since the S-protein is located on virion surface in the form of a homotrimer and the S1 subunit is more exposed than S2 [14, 18, 45], it is logical to assume that antibodies to the S1 subunit are driving antigenic drift of the virus, which results in some amino acids substitutions in this protein region [18, 19, 21]. Therefore, the antigenic determinants of the S-protein can no longer be recognized by antibodies produced against the previously circulating antigenic variant. The degree to which SARS-CoV-2 can evolve to evade neutralizing antibodies is a subject of active research [46].

It was shown that antibodies to N-protein of SARS-CoV-1 can neutralize the virus [47]. It is not yet clear if N-protein targeting antibodies can also neutralize SARS-CoV-2, however, highly significant correlations were found between the virus neutralization ability of COVID-19 patients’ serum and concentrations of antibodies targeting RBD or N-protein. In other words, both anti-N and anti-RBD IgG levels correlated with the virus neutralization titer and with each other [44]. Both types of antibodies, anti-N and anti-RBD, were produced in high titer by hospitalized COVID-19 patients [48]. Since N-protein sequence is more conservative compared to S-protein [21, 49] the protein can become an attractive vaccine target, if it will be confirmed that the protective antibodies can be formed against this protein in COVID-19 patients.

Off note: N-proteins of SARS-CoV, MERS-CoV and SARS-CoV-2 can bind to serine protease MASP-2, which participates in a complement activation pathway. The binding causes inflammatory lung injury in the mice model and therefore might cause similar problems in humans [50]. Perhaps the motif of the N-protein (115–123) that directly interacts with MASP-2 should be excluded from the future vaccine constructs [50].

### T-Cell Immune Response

The T-cell immunity might be extremely important for effective suppression of coronavirus infection. It is hypothesized that T-cell response against conservative antigenic determinants of seasonal (usually respiratory) coronaviruses, may prevent or mitigate SARS-CoV-2 infection [51]. This assumption is supported by the results of at least two research groups [52, 53], who found CD4+ and CD8+ T cells recognizing SARS-CoV-2 antigenic epitopes in a dozen healthy donors whose blood was collected before the COVID-19 epidemic. Among the recognizable epitopes were peptides from the proteins N, M, NSP3, NSP4, ORF3a, ORF8 [52] and/or N, ORF-1, NSP7 [53]. The effectiveness of the T-cell immune response to these epitopes in patients with COVID-19 was not lower compared to the response to S-protein epitopes [52]. It was shown that SARS-CoV-2 T cell immunity to the viral N-protein is comparatively strong [48] and is long lasting [53].

Being highly immunogenic and abundantly expressed during coronavirus infection N-protein [54] is much more conserved compared to S-protein between SARS-CoV-1 and MERS-CoV-1 viruses [49]. In addition, the N-protein induces long-lived memory T-cells after SARS infection in recovered patients [55]. However, some interaction properties of N-protein require caution in using it as a vaccine target [50].

Bioinformatics analysis of experimental data [56–58] identified evolutionarily conservative linear antigenic determinants of SARS-CoV-2, which can be used in the development of a vaccine that induces a T-cell immune response. Among these determinants are peptides of proteins N, M, and E [56]. Complete set of human linear antigenic determinants of SARS-CoV-2 recognized by immune system is available from the Immune Epitope Database (IEDB), which is a freely available resource funded by National Institute of Allergy and Infectious Diseases (USA).

Thus, we can conclude that SARS-CoV-2 has conservative antigenic determinants that can be used for design of a vaccine inducing a long-lasting cross-protective immune response against coronaviruses.

### The Mucosal Immune System in the Respiratory Tract

SARS-CoV-2 replication requires the cellular expression of angiotensin converting enzyme 2 (ACE2), which is a binding receptor for the viral S-protein [59]. The expression of proteases such as TMPRSS2 and furin is needed as well for proteolytic activation of the virus [15]. The cells expressing all three of these proteins are goblet cells in the nasal cavity, transient-type secretory cells located between the goblet and ciliary cells; type I and type II pneumocytes and small intestine enterocytes [60–62]. Recent data demonstrated that the virus could spread by bypassing the ACE2 receptor through S-protein mediated fusion of infected cells with uninfected ones. The infection spread via such a mechanism leads to the formation of multinucleated syncytia cells [63]. Cell fusion and the formation of syncytia facilitate the cell to cell spread of the virus and its escape from the host immune response [63]. The infection of immune cells by the ADE mechanism also cannot be ruled out—it may be the main mechanism that causes COVID-19 severe complications.

It should be noted that, COVID-19 disease can be accompanied by diarrhea [64]. The mechanism of infection of intestinal enterocytes with the coronavirus is not yet understood. Given that diarrhea is rare with COVID-19, it can be assumed that the main “entry gate” of the SARS-CoV-2 virus into the body is through the cells of the respiratory tract, which means that they should be the main targets when developing antiviral and prophylactic agents.

Taking into account that SARS-CoV-2 can spread from cell to cell by causing the formation of syncytium and without entering the intercellular space, it can be assumed that it encounters antibodies when it enters the bloodstream only at late stages of infection. It is worth noting that the presence of protective antibodies after vaccination in the blood, even with a high titer, may not be sufficient to inactivate the virus in the early stages of infection in the mucous membrane of the pulmonary epithelium.

In this regard, the vaccine should prevent the virus from entering the body through the main “entry gate” located in the epithelium of the mucous membrane of the upper respiratory tract before it enters the bloodstream. The vaccine should induce mucosal defense, i.e. the formation of primarily IgA antibodies and T‑cell response combination, which play a major role in mucosal immunity [65]. Thus a group of researchers has found that triggering antibody and cellular responses in the respiratory tract via intranasal vaccination against SARS and MERS might induce higher protection levels in mice [66, 67].

Creating a vaccine for respiratory pathogens that prevent infection at the virus entry site is a difficult task, but in the case of SARS-CoV-2 such a vaccine will be most effective.

## VECTOR VACCINES

One of the modern approaches to the development of vaccines is the expression of antigenic determinants of pathogen proteins in a vector construct based on a virus that is not pathogenic for humans. The advantage of this approach is its high immunogenicity and the ability to induce both cellular and humoral immunity against the pathogen.

Considering that the vector virus in the human body goes through a limited replicative cycle without developing an infection, it is clear that certain requirements are imposed on it, including the ability to express incorporated transgenes of pathogens in human cells and safety for humans. Such vector viruses are already known, such as the measles vaccine virus, vaccinia virus, adenoviruses, adeno-associated viruses, and others [68, 69].

China was one of the first countries to start developing vaccines against COVID-19. CanSino Biological Inc. (China), in collaboration with the Beijing Institute of Biotechnology, is developing a vaccine based on adenovirus serotype 5 (AdV5). Several other companies are also using AdV as a vector to create vaccines against COVID-19. For example, a large pharmaceutical company AstraZeneca (Great Britain), together with Oxford University (Great Britain), has started the third phase of clinical trials. Prior to this, this vaccine was tested in an animal model of primates [70]. A single dose of vaccine induced the production of antibodies in animals but did not provide 100% protection against infection. At the same time, experimental infection in the vaccinated monkeys was in a much milder form compared to the control animals which developed viral pneumonia after infection.

The use of AdV5 as a vaccine vector has a drawback. The human population already has antibodies to the virus proteins [71] and these so-called preexisting antibodies might reduce the effectiveness of vaccination. However, use of vectors that originated from adenoviruses of other than human species might solve this problem.

Institute Pasteur in France in collaboration with the Pittsburgh Center for Vaccine Research in the United States and Temis University in Australia, as well as Zydus Cadila, located in India are developing a vaccine using the measles vaccine strain as a vector. The attenuated measles virus as a vector base has already been used by scientists from Germany to create an experimental vaccine against MERS [72].

An interesting approach is taken by scientists from China, who use parainfluenza virus 5 [73] as a vector base. This virus, like the measles virus, belongs to the Paramyxoviridae family. It also has other names: monkey parainfluenza virus 5 and Simian virus 5 [74]. The parainfluenza virus 5 does not cause disease in humans, therefore antibodies to it are most likely absent in the human population. This means that parainfluenza 5 virus can be considered as a potential vector for vaccines.

### Sendai Virus as a Vector

COVID-19 is contracted by aerosolized virus-containing particles that penetrate the upper respiratory tract. Therefore SARS-CoV-2 is defined as a respiratory pathogen. Thus, a vaccine that induces mucosal long-term protection in respiratory airways would be highly valuable in controlling new epidemics. Sendai virus (SeV) also known as murine respirovirus represents an attractive candidate as a backbone for such a vaccine. Its main advantage compared to other vector candidates is its ability, to replicate in the cells of the human bronchial epithelium as well as in some categories of dendritic cells [75] without causing a disease [76]. The virus is also able to multiply in the cells of the bronchi of primates [77]. Thus, SeV is able to deliver both the viral antigens of the coronavirus and the product of its replication cycle, namely double-stranded RNA, which is a powerful pathogen-associated pattern that induces the body’s immune response, to both human bronchial epithelial cells and its dendritic cells.

It is extremely important that vaccines designed to protect against respiratory pathogens such as SARS-CoV-2 will be able to stop the infection spread in the mucosa of the bronchial epithelium of the upper respiratory tract (see section Localization of SARS-CoV-2 infection in the bronchial epithelium). Ideally, a vaccine would induce the production of IgA antibodies. A vaccine based on a viral vector that is itself a respiratory pathogen is best suited for this purpose since it can be used in the form of nasal drops.

SeV, a rodent pathogen has been known to the research community for almost 70 years. It has been widely used as a research tool in cell biology and in industry. Over the past three decades numerous genetically engineered SeV constructs, including vectors for foreign transgene delivery, have been created [78, 79]. SeV has several merits as a vaccine vector: the virus does not integrate into the host genome; it does not undergo genetic recombination with its host genes; and it replicates only in the cytoplasm without DNA intermediates or a nuclear phase.

Like all other representatives of the Paramyxoviradae family and negative sense RNA viruses, SeV is genetically stable and evolves very slowly [80]. It belongs to a category of viruses that are governed by the “rule of six” [81–83]. Like the genomes of other paramyxoviruses, SeV usually includes six genes, which encode six major proteins. The low rate of homologous RNA recombination in paramyxoviruses genomes probably results from the unusual genomic requirement for polyhexameric length (6n+0) [83]. Therefore, SeV infection provides a stable foreign gene expression system.

**Fig. 4.**
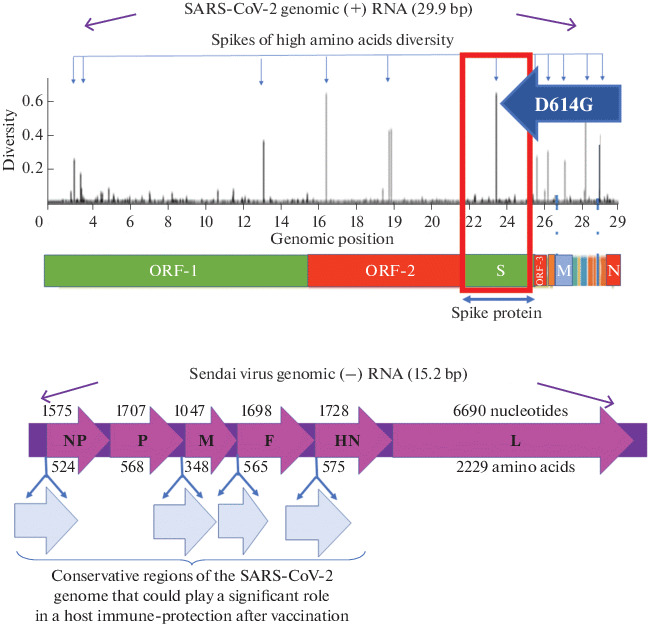
The organization of the genomes of the SARS-CoV-2 and Sendai viruses. Above—the genome of SARS-CoV-2 virus and the amino acids variability histogram. The variability scale shows the proportion of non-identical amino acids in each position in the database, collected from December 2019 to June 2020 [97] and includes 2921 viral variants. The first amino acid sequence of proteins encoded by the SARS-CoV-2 genome, published in the database, was taken as a standard. The variability of the amino acid at each position was calculated as the proportion of amino acids found in the database that were not identical to the reference one for all proteins in the translated part of the genome. Below is the genome of the Sendai virus and potential sites for the transgene’s introduction [56, 57]. Transgenes may encode conservative antigenic determinants of SARS-CoV-2 [41, 44, 98]. The encoded proteins are indicated in Latin letters. The numbers that are above the scheme of the Sendai virus genome indicate the length of individual genes, and numbers below—the size of the corresponding proteins.

The SeV genome is negative-sense RNA, non-segmented, about 15384 nt in length. It contains six cistrons and noncoding 3' leader and 5' trailer regions, which are each about 50 nucleotides in length [78, 84]. This simple genome structure has encouraged many researchers to construct a recombinant SeV vector by adding foreign genes or replacing viral F, HN and M genes [78, 79, 84–90]. It has been demonstrated that a gene of more than 3 kb can be inserted and expressed in SeV [84]. Foreign genes can be incorporated into the SeV genome at multiple positions, among them the noncoding 3' leader region before the NP gene [91], the region between the F and M genes [92], the region between the F and HN genes [93], and the 3' noncoding region of the P gene [94]. A SeV vector backbone that incorporates conservative immunogenic elements of SARS-CoV-2 genome [56] has a high likelihood to generate a safe and immunogenic vaccine (Fig. 4).

An additional advantage of SeV as a vaccine vector candidate is that it could be delivered in a form of nasal drops [85]. This type of delivery also induces long-term protection in mucosal respiratory airways. Moreover, intranasal administration reduces the effect of a pre-existing immunity to SeV, as compared to intramuscular delivery [95]. Replication competent SeV has been used in clinical trials involving both adults [96] and children [85] to immunize against human parainfluenza virus type 1 (HPIV-1). Virus administration in the form of nasal drops in doses ranging from 5 × 10^5^ to 5 × 10^7^ 50% embryo infectious doses (EID50) induced the production of neutralizing antibodies to the human virus without any measurable side effects [85]. The results of these trials represent evidence of SeV safety for humans.

It has been also shown in preclinical research [86] and in clinical trials [99] that SeV promotes high levels of antigen specific CD8+ T-cell responses. The development of an AIDS vaccine with a SeV vector is in phase II clinical trials. Evaluation of the safety and immunogenicity of an intranasally administered replication-competent SeV—vectored HIV Type 1 gag protein vaccine demonstrated induction of potent T-cell and antibody responses in prime-boost regimens [86, 99]. SeV has also been used as the backbone for vaccines against tuberculosis [87, 100] and respiratory syncytial virus (RSV) [88, 89]. RSV, also called human orthopneumovirus, is a major cause of lower respiratory tract infections and hospital visits during infancy and childhood. Vaccine development against RSV is in a phase I clinical trial [88].

SeV antibodies that cross-react with HPIV-1 antibodies are present in most people, but in low titers. A study published in 2011 demonstrated that SeV neutralizing antibodies (formed due to HPIV-1 past infection) can be detected in 92.5% of subjects worldwide with a median EC50 titer of 60.6 IU/mL [101]. It is believed that this low titer is not an obstacle to efficient vaccination with SeV vectors because a low background of anti-SeV antibodies does not block the ability of an SeV-based vaccine to promote antigen-specific T cell immunity [102].

Additional advantages of SeV as a vaccine vector also include high productive capacity in specific pathogen-free embryonated chicken eggs [103]. Moreover, SeV can be adapted to grow in FDA-approved mammalian cell lines. Multiple rounds of directed evolution increase the virus titer in various cell cultures [104–106].

Availability of a reliable animal model is an important factor for developing a successful vaccine. One potential problem in using SeV as a vaccine vector in a mouse model is susceptibility of mice to infection triggered by the virus. However, some mouse strains such as C57/6J are comparatively resistant to the virus and recover after an infection. These mice can tolerate a high infectious dose (10^5^ EID50) of SeV [90, 107]. F344 rats are also SeV-resistant [108]. A SARS-CoV-2 infection model in mice was recently created [109]. Thus, humoral, and cellular immune responses of the vaccine candidate most likely could be evaluated in a mouse animal model.

In China, Fudan University in collaboration with Pharma Co. Ltd. is engaged in development of a vaccine for COVID-19 prevention. Replication deficient SeV serves as a backbone vector in the project. However, a replication competent strain also can be used for vaccines [85, 96, 99]. Both types of vectors, replication competent and replication deficient, have their pros and cons. They have never been compared side by side in terms of immunogenicity and safety in preclinical studies or clinical trials. Nevertheless, it is worth mentioning that the replication competent vector is safe and does not cause serious side effects even in children [85]. Moreover, it is easier and cheaper to produce because it requires fewer preparation steps.

We believe that the SeV vector backbone has the potential to generate a successful COVID-19 vaccine with enormous global health benefits.

## CONCLUSIONS

We assume that viral infection of immune cells by the ADE mechanism can occur both in the case of a severe course of the COVID-19 disease after primary viral infection, and in case of re-infection with an already antigenically modified strain. Immunization against SARS-CoV-2 can aggravate subsequent infection, and this possibility must be considered, both when studying the pathogenesis of COVID-19, and when predicting the next waves of the epidemic. Attracting attention to the ADE phenomenon, its mechanism and modeling is very important now that trials of vaccines against COVID-19 are already going on with full speed ahead.

High variability of S-protein glycosylation patterns along with this protein conformational mobility all promote antigenic diversity of SARS-CoV-2 isolates. This diversity makes this protein a non-optimal vaccine target antigen because it can promote ADE or/and short-lived vaccine related protection.

It is likely that the use of conservative antigenic determinants of N-protein in the SARS-CoV-2 vaccine instead of the rapidly changing S-protein determinants will, firstly, achieve a long-term immune response to this pathogen and, secondly, reduce the likelihood of ADE development.

All genes for proteins encoding conservative antigenic determinants of the virus can be incorporated into constructs based on the Sendai virus vector platform.

The vector platform based on this virus has several advantages. The virus is characterized by high genomic stability and safety for humans. In addition, vaccines based on respiratory viruses, the category of which includes the Sendai virus, can induce mucosal immunity, and prevent viral infection in the mucous membrane of the respiratory tract, the “gateway” of the SARS-CoV-2 pathogen. Thus, by incorporating the conservative antigenic determinants of SARS-CoV-2 into the genome of the Sendai virus, a vaccine construct can be created that will provide effective and long-term protection against COVID-19.

## ADDITIONAL INFORMATION

The first two authors have contributed equally to the work.
